# Hijackers on the open access highway

**DOI:** 10.1002/nop2.36

**Published:** 2015-10-14

**Authors:** Roger Watson

First some good news: *Nursing Open* is now listed in the Directory of Open Access Journals (DOAJ) which means that people considering submitting to *Nursing Open* can be confident that we are a *bona fide* open access journal and that we meet the standards required to be listed there, one of which is that we have had to be publishing for over one year. In fact, we are now well into our second year and I am very happy to bring you our third issue which brings us to a total of nearly 30 papers published since we were launched in 2014. It is also busy at the editorial desk and each week brings a flow of new submissions and I send a steady flow of manuscripts to production. Few things are more satisfying than correcting proofs for *Nursing Open* and our Twitter following @nursingopen is increasing as are entries and hits on the *Nursing Open* blog.

## Hijackers

I also edit *Journal of Advanced Nursing* (*JAN*) and a recent submission to our *JAN interactive* blog is relevant to *Nursing Open* and our potential authors and readers. The entry was by Mehdi Dadkah, Foulad Institute of Technology, Tehran, Islamic Republic of Iran – it is titled: ‘More on the dark side of open access’ – and it deals with the issue of online open access journal hijacking. I was aware of the problem but had not seen a statement on it and I now realize that journal hijacking is systematically studied by Mehrdad Jalalian and it is a worrying phenomenon. The online open access movement has been full of good intentions and there are many examples of successes and many examples of legitimate journals, as shown by the DOAJ. However, the online environment is malleable, inexpensive and wide‐reaching, and criminal elements have been quick to exploit the pressure on academics to get published. Witness the rapid rise in predatory publishers and the work of Jeffrey Beall which the editorial team of *JAN* recently wrote about (Pickler *et al*. 2015) and which *JAN* also commented in the early days of online open access publishing (Watson *et al*. 2012). Essentially, predatory publishers are those who prey on academics through email bombardment with seemingly legitimate names and flattering invitations to publish – usually with minimal editorial interference – and for seemingly reasonable prices.

Superimposed on the problem of predatory journals is the phenomenon of hijacked journals. For the recipient of the perpetual stream of open access email invitations, it can be hard to discern predatory from legitimate journals, and predatory and legitimate journals from hijacked journals. Hijacked journals are those that appear to be legitimate – with identical or similar names to *bona fide* journals – but are, in fact, fake. They take money for publishing an article with no intention of publishing it and then disappear from the online environment. The mechanisms vary but one example I have seen is an invitation to publish in the *Journal of Advancing Nursing* which had a website that was, superficially, like that of *JAN*. I had no evidence to show my publisher and have no evidence now as the website quickly disappeared. Presumably they had existed long enough to convince a few people to part with money with the promise of publishing their article. However, it also appears that it is possible to hijack genuine journal webpages but still to convince naïve authors to part with money.

## Advice

As the editor of a legitimate DOAJ listed online open access journal, these examples of fraudulent activity concern me. They bring the whole field of online open access publishing into disrepute and they part naïve but well‐meaning authors from their money. Is there anything that authors can do to avoid being defrauded? I believe that some simple rules are sufficient, as follows: If you are invited by email to submit to online open access journals, firstly, check whether the journal is listed in (DOAJ). If it is, does it have a tick next to it to show it has met the DOAJ criteria for acceptance? If it is not, Google the journal webpages and submission systems: does the journal follow COPE guidelines and have set policies in place in relation to research ethics? Does the journal publish content regularly? Is there editorial independence? Does the journal have an established editorial team of more than one Editor and a well‐represented regional Editorial Board? Do you recognize any of the names associated with the journal? If you do not recognize any names, or the people do not appear to work in the purported area of the journal or they all come from one country, then it is best to avoid the journal. If you do recognize names then check with them that they are legitimately listed as it is also the case that individuals are hijacked to lend bogus journals credibility, and can have difficulty having their names dissociated from the journal.

## Conclusion

Online open access journals in all fields, including nursing, are growing in number and gaining credibility. However, danger lurks along the way for unwary authors. There are some obvious signals that can reveal predatory or hijacked journals and it is possible to check online to verify the status of any journal which contacts you. But the best advice I can offer you is, when you receive an email from an online journal inviting you to submit: if in doubt, do not!
